# Ashtanga-Based Yoga Therapy Increases the Sensory Contribution to Postural Stability in Visually-Impaired Persons at Risk for Falls as Measured by the Wii Balance Board: A Pilot Randomized Controlled Trial

**DOI:** 10.1371/journal.pone.0129646

**Published:** 2015-06-24

**Authors:** Pamela E. Jeter, Steffany Haaz Moonaz, Ava K. Bittner, Gislin Dagnelie

**Affiliations:** 1 Department of Ophthalmology, Lions Vision Research Center, Wilmer Eye Institute, Johns Hopkins University, Baltimore, Maryland, United States of America; 2 Department of Integrative Health Sciences, Maryland University of Integrative Health, Laurel, Maryland, United States of America; 3 College of Optometry, Nova Southeastern University, Ft. Lauderdale, Florida, United States of America; University of Toronto, CANADA

## Abstract

**Objective:**

Persons with visual impairment (VI) are at greater risk for falls due to irreparable damage to visual sensory input contributing to balance. Targeted training may significantly improve postural stability by strengthening the remaining sensory systems. Here, we evaluate the Ashtanga-based Yoga Therapy (AYT) program as a multi-sensory behavioral intervention to develop postural stability in VI.

**Design:**

A randomized, waitlist-controlled, single-blind clinical trial

**Methods:**

The trial was conducted between October 2012 and December 2013. Twenty-one legally blind participants were randomized to an 8-week AYT program (n = 11, mean (SD) age = 55(17)) or waitlist control (n=10, mean (SD) age = 55(10)). AYT subjects convened for one group session at a local yoga studio with an instructor and two individual home-based practice sessions per week for a total of 8 weeks. Subjects completed outcome measures at baseline and post-8 weeks of AYT. The primary outcome, absolute Center of Pressure (COP), was derived from the Wii Balance Board (WBB), a standalone posturography device, in 4 sensory conditions: firm surface, eyes open (EO); firm surface, eyes closed (EC); foam surface, EO; and foam surface, EC. Stabilization Indices (SI) were computed from COP measures to determine the relative visual (SI_firm_, SI_foam_), somatosensory (SI_EO_, SI_EC_) and vestibular (SI_V_, i.e., Foam_EC_ vs. Firm_EO_) contributions to balance. This study was not powered to detect between group differences, so significance of pre-post changes was assessed by paired samples t-tests within each group.

**Results:**

Groups were equivalent at baseline (all p > 0.05). In the AYT group, absolute COP significantly increased in the Foam_EO _(t(8) = -3.66, p = 0.01) and Foam_EC_ (t(8) = -3.90, p = 0.01) conditions. Relative somatosensory SI_EO_ (t(8) = -2.42, p = 0.04) and SI_EC_ (t(8) = -3.96, p = 0.01), and vestibular SI_V_ (t(8) = -2.47, p = 0.04) contributions to balance increased significantly. As expected, no significant changes from EO to EC conditions were found indicating an absence of visual dependency in VI. No significant pre-post changes were observed in the control group (all p > 0.05).

**Conclusions:**

These preliminary results establish the potential for AYT training to develop the remaining somatosensory and vestibular responses used to optimize postural stability in a VI population.

**Trial Registration:**

www.ClinicalTrials.gov NCT01366677

## Introduction

As of 2010, there is an estimated 3 million cases of vision impairment (VI) in the U.S. in persons 40 years and older and this number is expected to increase dramatically to approximately 7 million by 2030 due to the growing aging population[1]. As the population ages, the elderly experience a loss in strength, sensorimotor processing, increased reaction times, and reduced vision[2,3]. Loss of visual function (e.g., visual acuity, visual fields) due to common age-related disease, such as macular degeneration, glaucoma, and cataracts, reportedly doubles the risk of falls[4] and is significantly associated with fall-related injuries leading to decreased mobility, independence and quality of life[5–11]. In a recent prospective, observational study, over half of new patients seen in the low vision rehabilitation service (n = 564; median age = 77) reported having a fall, and of those, 39% reported that the fall was vision related[12].

The postural control system consists of the musculoskeletal, sensory, and central nervous systems working together to maintain postural stability[13]. To avoid falls, the central nervous system continuously monitors feedback from the active sensory systems to generate corrective musculoskeletal responses that regulate postural control[14,15]. Specifically, sensory input is derived from three different sources that operate synergistically: somatosensory (e.g., proprioception), vestibular (e.g., changes in head position), and the visual system (e.g., optic flow, visual fields)[16–19]. Thus, individuals with uncorrectable vision loss have a damaged visual input stream, contributing to postural instability[20]. Research into the role of each sensory system under experimental or pathological conditions during standing balance has led to a greater understanding of how the remaining sensory inputs can adjust when one system is impaired[21–25]. Individuals with a compromised visual contribution to balance are even more unstable in conditions when the somatosensory system is also perturbed[16,17,21,24,26,27], e.g., when walking on thick carpet with thick-soled shoes or on a sandy beach. Similar instability patterns have been observed for cases in which vision and vestibular systems are impaired[21,23].

An important property of the postural control system is its ability to “down-weight” poor sensory cues and “up-weight” more reliable cues, also known as sensory reweighting[15,28]. The ability to reweight sensory inputs might suggest that VI individuals could make better use of the remaining sensory inputs upon losing vision, but this may not happen without specific training[25,29,30]. Targeted balance training may significantly improve postural stability by strengthening or “up-weighting” the remaining sensory systems[15,29,31–34]. For example, training that stimulates the use of postural control strategies, such as the ankle and hip adjustments, can subsequently stimulate the somatosensory and vestibular systems[31,32,35–37].

Real-world falls are difficult to measure, therefore, center of pressure (COP) has been used as a surrogate marker for postural stability in standing balance[38–41], especially since it is correlated with sensorimotor deficits[42]. COP component measures quantify the displacement of upright balance around the center of mass through the distribution of total force applied to the supporting surface[43]. Using a clinically validated balance protocol and a force platform[44] one can derive measures of absolute COP and easily quantify the relative visual, somatosensory and vestibular contributions to balance by artificially removing one or more conditions and calculating stability indices[18,24]. When postural stability (i.e. quiet standing) is perturbed due to unexpected environmental changes, a proportionate increase in COP magnitude is observed, therefore, a reduction in absolute COP displacement would imply greater postural stability[45]. For example, COP displacement is augmented during walking or during changes in sensory input[46]; similarly, when measured at baseline, VI individuals show greater COP displacement when compared to normally sighted individuals[16,24,26,27]. Normally sighted individuals show less “movement”, thus, greater postural stability. Therefore, COP minimization as an indicator of greater stability and reduced fall risk is often cited in the literature[43].

On the other hand, multiple studies have suggested that minimization of COP parameters may not be the best indicator of better stability, in particular for clinical populations. For example, COP can be reduced in clinical populations at risk for falls such as Parkinson’s patients, lower leg amputees and ACL-deficient individuals, compared to healthy, normal adults[41,47,48]. In addition, absolute COP and relative sensory contribution to balance can be increased after significant balance training, such as Tai Chi and other forms of exercise[33,49–52]. As long as balance is maintained safely within the individual’s base of support and sensorimotor feedback is augmented in order to compensate for sensory deficiencies via corrective strategies, the increase in stability recorded as greater COP displacement, is then considered an indicator of COP stabilization[28,43,48]. Therefore, COP minimization may not apply to systems that are inherently unstable (e.g., musculoskeletal disorders, elderly)[28]. While the mechanism for COP changes after training is not clear, it is possible that individuals develop corrective strategies to down-weight unreliable and up-weight reliable information[28]. For example, increased flexibility in the ankle joint may provide meaningful proprioceptive feedback to generate adaptive motor responses for improved posture[53,54].

Yoga, a popular wellness activity, is a strong candidate for therapeutic intervention since it provides an integrated, multisensory approach that can engage the use of compensatory sensory inputs. For example, emphasizing firm foot placement while maintaining balance may activate the somatosensory system, thereby generating proprioceptive learning. An increase in proprioceptive joint sense in the elbow has been observed for congenitally blind children after one month of yoga training[55]. Safely executing movement in standing or semi-inverted postures activates the vestibular system[56,57]. One study investigated highly trained yoga practitioners and found them more reliant on internal vestibular and proprioceptive signals than external visual cues in a multisensory integration perceptual task[58]. In another study, yoga participants were more able to retain balance on a vertical force platform in an eyes-closed condition compared to controls, indicating more effective use of proprioceptive cues[32]. Balance improved substantially in a separate study using a timed, one-legged balance test after yoga training in healthy, younger adults[59] and in normally sighted, older population[60]. Yoga has been used in clinical settings to improve balance and posture for patients with osteoporosis[61]. Studies have suggested that yoga may work by strengthening muscular endurance, proprioceptive awareness and by cultivating better breath control, which may facilitate steadiness and awareness in balance[59,62]. Thus, targeted training, such as yoga, may significantly improve postural stability by strengthening the remaining sensory systems in our VI population.

The Ashtanga-based Yoga Therapy (AYT) is a highly modified Ashtanga yoga sequence developed specifically for the VI population by the author (PEJ)[63]. Ashtanga is a system of yoga taught by Sri K. Pattabi Jois in Mysore, India. It is an integrated system of *asanas* (postures), *vinyasa* (movement), and *ujjayi* (victorious breath). Importantly, Ashtanga emphasizes the use of core locks called *bandhas*, prominently located at the root of the spine and lower abdomen comprising the pelvic area. Learning to engage these areas through standing and hip opening postures with coordinated movement may strengthen muscle and increase flexibility. Thus, the AYT sequence of postures was selected to promote balance with a focus on strength, hip opening (e.g., adduction, abduction) and ankle flexibility (e.g., dorsiflexion)[53,57,60]. While the sequence of *asanas* remains the same each session, each *asana* is modified to suit the individual’s needs, and can be completed regardless of age, fitness, or level of experience. The feasibility of the AYT was evaluated in a previous pilot study and found to be suitable for a VI population since poses can be easily articulated verbally[64]. In the previous pilot study, three participants who completed the timed one-leg stand (OLS) developed an increase in static balance; however, changes in sensory contributions after AYT have yet to be evaluated.

The objective of the present study was to examine the influence of an 8-week AYT program on COP for VI individuals compared to a waitlist control. We introduce the novel use of a cost-friendly, readily accessible Wii Balance Board (WBB; Nintendo, Tokyo, Japan), an accessory to the popular Wii Fit video gaming system, as a standalone posturography device to quantify COP during static balance conditions[27]. Specifically, we evaluated the results in the context of a COP stabilization or minimization framework. We hypothesized that COP displacement may increase, reflecting subtle adjustments in muscle control to achieve greater stability after AYT, in support of stabilization. We further hypothesized that AYT will develop the relative contributions of somatosensory and vestibular systems to postural stability.

## Methods

### Trial Design and Ethics Statement

We conducted a randomized, waitlist-controlled pilot study with VI individuals from the Maryland, Virginia, and Washington D.C. area. The study protocol (Protocol # NA_00039032) was approved by the Institutional Review Board of the Johns Hopkins University School of Medicine, followed the tenets of the Declaration of Helsinki and is registered at www.clinicaltrials.gov (NCT01366677) .The approved protocol for this trial and supporting CONSORT checklist are available as supporting information; see S1 Protocol and S1 CONSORT Checklist. Initial screening was conducted over the phone. Due to transportation limitations, all assessments were conducted at the Lions Vision Center or the studio locations (a benefit of using the WBB). All participants provided written informed consent. After baseline assessments were completed, participants were randomized to either an 8-week AYT program or waitlist control group. Randomization to group assignment was conducted by the study PI using the random number generator in MATLAB (Mathworks, Inc.). A research assistant assigned unidentifiable subject IDs (i.e. #1–21) to subjects after enrollment. Numbers (i.e. #1–21) were randomly ordered in MATLAB and assigned to the randomly ordered anonymous subject IDs. Subject IDs that were paired with even numbers were assigned to the intervention and those paired with odd numbers were assigned to the waitlist control. Masking participants to the yoga intervention was not possible. After the initial AYT group completed the program and assessments were collected, the waitlist groups were invited to participate in the AYT program at no cost. To make the study more accessible to VI participants, the AYT program was conducted at studio locations in the Washington DC metro area, and participants were reimbursed for public transportation if needed. The multiple locations also allowed us to facilitate teacher-student interaction with smaller class sizes. Treatment fidelity was ensured as described below.

### Deviations from study protocol

The trial protocol was written in a general format to address several research questions. An exploratory study [64] was conducted previously to determine feasibility of the AYT and details preliminary findings, which informed the current study. The study presented here evaluates the hypotheses that absolute COP and the relative contribution of remaining sensory inputs to postural stability increases after an 8-week AYT program. Therefore, only objective measures of COP are presented, in addition to a clinical balance and physical fitness assessment. These data represent the quantitative results as a subset of a larger battery of assessments that included psychological questionnaires and other qualitative information. The latter have been presented in preliminary form[65] and will be the subject of a separate manuscript presented elsewhere.

**Specific deviations from the protocol include the following:**
- Study location -- The yoga study was not conducted at the Lions Vision Center as originally planned when the protocol was written due to the space no longer being available. Furthermore, our study population faces considerable transportation challenges and so, the study took place at local studio spaces in the Washington DC metro area to accommodate our study participants’ accessibility needs.
- Baseline measures—Only measures that pertained to the current research question regarding risk factors for falls were measured. Clinical vision assessments (e.g. visual acuity) were not conducted at screening due to limitations in resources and accessibility. Obtaining recent eye exams with the study patient’s consent to determine legal blindness eligibility was sufficient and less resource intensive.
- Center of pressure—Several parameters can be derived from COP data, each of which describes changes in postural stability[43]. In a previous study[27], we determined the test-retest reliability of all COP parameters using the WBB in a VI population. Mean Total Velocity (MTV) (mm/s) was found to be the most consistent and reliable variable as a two-dimensional representation of postural stability in the anterior-posterior and medial-lateral direction. Therefore, MTV (mm/s) was derived from COP data for analysis, instead of root mean square of COP amplitude listed in the protocol. Furthermore, MTV has been shown to accurately represent the visual contribution to posture[18,26].
- Statistical Analysis and Sample Size -- A sample size calculation was not applicable to this type of exploratory, pilot study, however, a minimum of 10 subjects per group was feasible based on our previous study to provide an indication of the acceptability of the AYT intervention[64] and to help suggest possible benefits across a moderate-sized group of visually-impaired subjects. The results provided here will allow us to further develop the intervention protocol and plan for larger scale RCTs as indicated in the post-hoc calculated sample size presented in the Results section. Finally, this study was not powered to detect between group differences following the AYT using an ANCOVA, so significance of pre-post changes was assessed by paired samples t-tests within each group for all outcomes.

### Participants

**Fig 1:**
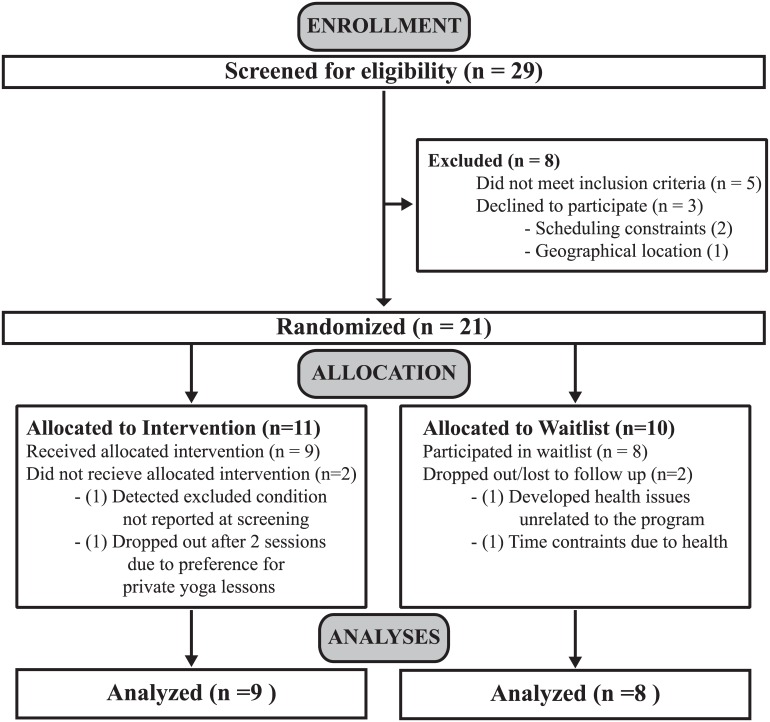
Participant Flow Chart.

Participants were recruited from the Low Vision Clinic of the Wilmer Eye Institute at Johns Hopkins Hospital (Baltimore, MD) and local advocacy groups (e.g., Foundation Fighting Blindness Maryland Chapter, Northern Virginia Chapter of the American Council for the Blind) through email and flyers to prospective participants. Recruitment began in October 2012 and the baseline assessments were completed in April 2013 (Fig 1). Inclusion criteria for VI participants were: (A) greater than 18 years of age, (B) legal blindness (best corrected visual acuity worse than 20/200 and/or visual field less than 20° in diameter, in the better eye) as determined by medical records, (C) any ocular disease that is expected to remain relatively stable throughout a 3–6 month period, (D) healthy to the extent that participation in a yoga program would not exacerbate any existing disease conditions, and (E) English spoken as the primary language. Participants were naïve to yoga or had not participated in a yoga class in at least two years. Exclusion criteria included individuals with vestibular disorders, acute orthopedic problems that affect ambulation, history of neurologic disease (e.g., peripheral neuropathy), or who were pregnant or taking medication that could affect balance (e.g., sleeping pills).

While postural stability is known to deteriorate with normal aging, VI may accelerate these effects[4]. A study by Era et al.[66] provides normative data from force platform tests for a cohort of 7979 healthy, sighted people ages 30 and above. Balance function begins to deteriorate at middle age (> 40 years and < 60 years old) and accelerates after age 60. In a VI population, another study indicated that injurious falls in persons less than 60 years old was associated with loss of visual function (i.e., visual acuity loss) and for persons over 60 years old, fall injury was more prevalent and also associated with poor contrast sensitivity[5]. Given the evidence that individuals may experience postural instability during a wide age range, not just > 60 years old, and considering the pilot nature of this study we did not exclude participants on the basis of age.

### Intervention

Participants took part in an orientation session before the yoga program began, in order to familiarize them with the style of yoga, the *ujjayi* breathing technique, and alignment techniques using the mat, class etiquette, and modifications. AYT subjects convened for one group session per week with the instructor (PEJ) and an experienced yoga assistant. Participants were provided with a free yoga mat and an audio CD developed by the author (PEJ) to practice at home and were asked to practice at least twice a week (i.e. equivalent to approximately 16 home practice sessions during the intervention period). Teaching yoga to those with VI requires simple modifications to postures, clear descriptions, and hands-on adjustments[64]. Each class began with simple seated breathing, a warm-up, standing postures, seated postures, followed by breathing and a final resting pose. Table 1 lists the full sequence of poses taught during AYT. The AYT is amenable to study because it is composed of a standardized sequence of postures held for a fixed duration. Each pose was held for five breaths or for as long as the subject was able. Each class included a question and answer period at the beginning and end of class.

**Table 1:** Ashtanga-based Yoga Therapy Sequence.

Time	Order	Poses
15 mins	1	*Padmasana* “Lotus Posture” or comfortable seated position and commence *ujjayi* breathing. (25 Breath Count, 5 minutes)
	2	*Moving with the breath—*From seated position, inhale the arms overhead drawing the palms toward each other until they touch. Exhale releasing the arms down moving with the breath. 5x
	3	Transition to Table Pose
	4	*Marjaryasana* (Cat) to *Bitilasana* (Cow)
	5	From Table to *Balasana* (Child's) pose
	6	From Table to Downward-facing Dog. Move from Downward-facing dog to standing.
30 mins	7	*Tadasana* or *Samasthiti* (Mountain Pose)
	8	*Padangusthasana* (Foot Big Toe Posture)
	9	*Utthita Trikonasana* (Extended Triangle Posture)
	10	*Utthita Parsvakonasana* (Extended Side Angle Posture)
	11	*Parivritta Parsvakonasana* (Revolved Side Angle Posture)
	12	*Prasarita Padottanasana*, A-D (Feet Spread Intense Stretch Posture)
	13	*Vrksasana* (Tree pose)
	14	*Utkatasana* (Chair Pose)
		*Move to the floor*, *modified Sun Salute*
	15	*Dandasana* followed by *Paschimottanasana* (Western Intense Stretch Posture)
	16	*Purvottanasana* (Eastern Intense Stretch Posture)
	17	*Janu Sirsasana* A (Head to Knee Posture)
	18	*Marichyasana* A (Dedicated to Marichi, son of Brahma)
	19	*Marichyasana* C (Dedicated to Marichi)
	20	*Navasana* (Boat Posture)
	21	*Baddha Konasana*, A &B (Bound Angle Posture)
	22	*Setu Bandha Sarvangasan*a (Bridge pose)
15 mins	23	*Paschimottanasana* (Western Intense Stretch Posture)
	24	*Padmasana* (Lotus Posture) or *Sukhasana* (Easy Pose), breathe 10x
	25	*Savasana* (Corpse Pose) (5–10 minutes)

### Fidelity of Treatment

Uniformity in AYT implementation was employed at the yoga studios to administer the same intervention to all participants. Several steps were taken to standardize the delivery of the intervention and evaluate its fidelity to the protocol.

#### Training

Author (PEJ), a trained Ashtanga teacher of four years and practitioner of 10 years, delivered the AYT protocol to all participants during the 8-week intervention, with the hands-on assistance of a trained Ashtanga yoga assistant.

#### Treatment Fidelity

Two research assistants took turns conducting visits to the yoga studios to monitor and observe the fidelity of the intervention administration to the protocol. No deviations from the protocol were observed during four visits.

#### Class Attendance

The AYT instructor monitored class attendance carefully for each participant. The AYT instructor was available for questions and answers before and after the class, and communicated with the yoga assistant and research assistant for any concerns regarding compliance.

#### Home Practice Logs

Participants were asked to complete a weekly practice log to report the activities performed, adherence to the CD, and time allotted to the practice, in order to help evaluate compliance. A research assistant collected the logs on a weekly basis.

### Outcome Assessments

All participants completed outcome assessments after the intervention group completed 8-weeks of AYT. Masking participants to the intervention was not possible, however, trained research assistants were masked to the group assignment during data collection post-AYT. Objective measures of balance are reported here, but were a part of a larger battery of tests that included psychosocial and qualitative measures, to be reported elsewhere.

### Primary Outcomes

#### Center of Pressure

The WBB was used as a standalone posturography device to measure COP, a marker for postural stability, while participants performed a validated, standardized static balance protocol[27]. Mean Total Velocity (MTV) (mm/s) was calculated as the total distance traveled by COP over time and used as the primary outcome, since it has been shown to accurately represent the visual contribution to posture[18,26].

**Fig 2:**
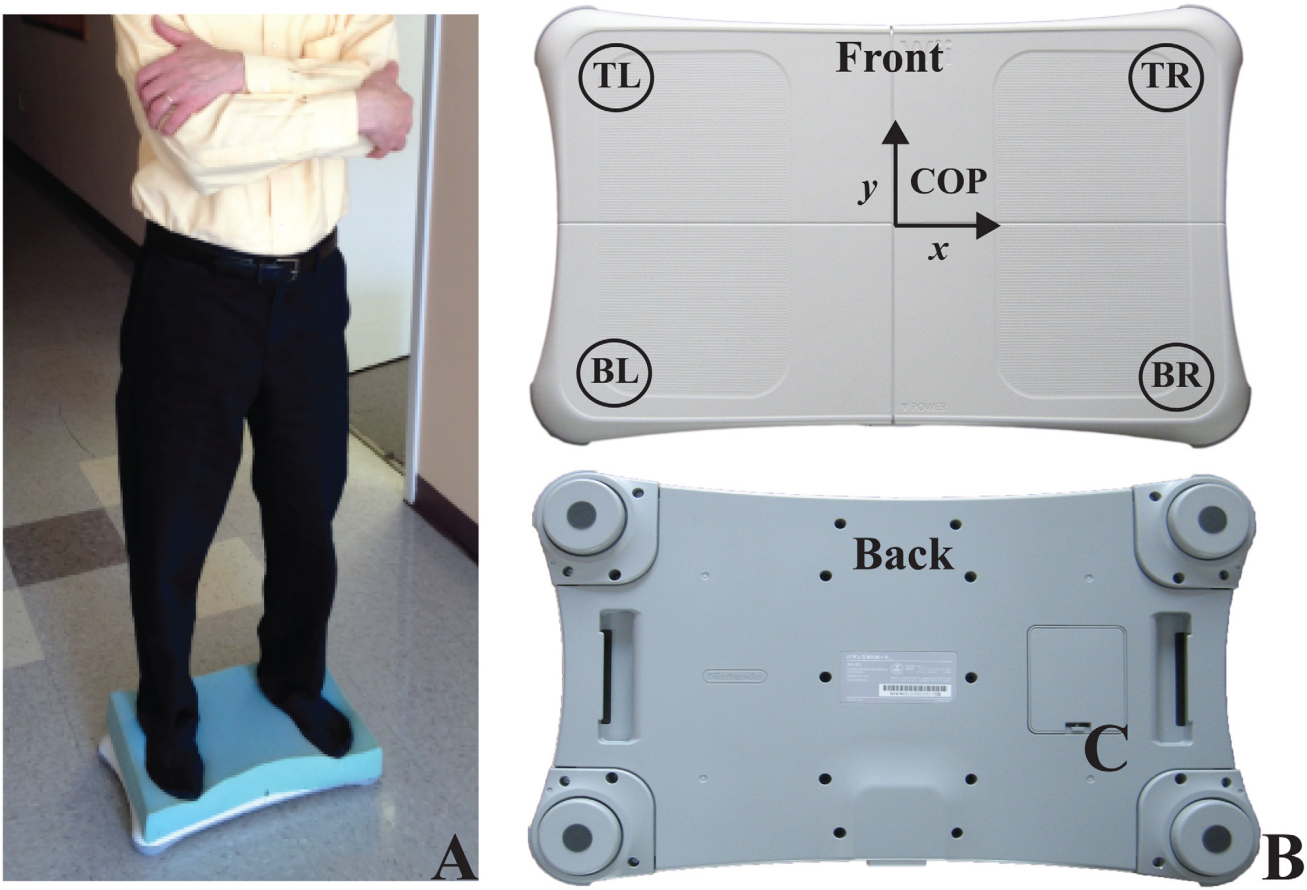
Use of the Wii Balance Board for the mCTSIB protocol. A) Subject standing on foam, B) Wii Balance Board. Four sensors are located in each corner. Center of Pressure is derived as an *x* and *y* coordinate measured over time.

The modified Clinical Test of Sensory Interaction in Balance (mCTSIB) was used as the measurement protocol to quantify how well participants used sensory inputs: somatosensory (firm vs. unstable surface), visual (eyes open vs. closed), and vestibular (stable surface with eyes open vs. unstable surface with eyes closed)[44]. COP was measured for each mCTSIB condition. The participant’s bare feet were placed on the WBB so that the inner edges of both feet were one-foot length (their own) apart, in four sensory conditions increasing in difficulty. The distance between the inner edges of the feet was divided in half and the subject was aligned accordingly with the halfway mark on the center of the WBB for all conditions during baseline and post-AYT. The feet position provides a wider base of support for our visually impaired study population in order to help promote safety in the foam condition and was chosen based on previous literature involving elderly patients with vision loss[16,26,27]. While the classic Romberg test (i.e., feet together)[67] is the most well-known, a study designed to determine normative values found that the Romberg test may be too variable for subjects older than 50 years old[68]. The conditions were: standing on a firm surface with eyes open (Firm-EO); standing on a firm surface with eyes closed (Firm-EC); standing on an unstable (3” thick[44]) surface with eyes open (Foam-EO; Fig 2A); and standing on an unstable surface with eyes closed (Foam-EC). Thus, conditions with eyes closed disrupted the visual system, whereas conditions on Foam disrupted the somatosensory system. It is important to note that the VI participants in this study had varying degrees of remaining vision that could contribute to, or interfere with, posture stabilization; therefore, comparing the eyes open and closed conditions allowed us to determine visual dependency[17]. The conditions within the balance tests were randomly ordered in both sessions. Prior to each condition, participants had a 30-second familiarization period to reduce learning effects. Participants performed each condition for 3 successive trials of up to 30 seconds each, with a one-minute rest period in between. MTV was calculated from COP data and was averaged across 3 trials. In order to minimize the influence of the vestibular system, participants were instructed to maintain a neutral head position, and when possible, to binocularly fixate on a black “X” taped on a white wall at eye level 35 inches in front of them while keeping their arms crossed over the chest. Target distance was kept constant at 35 inches, a distance shown to minimize the possible influence of visual input on postural stability[26,69]. Thus, the level of visual impairment should not influence postural stability.

#### Stability Index

The MTV COP measures under the four mCTSIB conditions were used to compute Stability Indices (SI), representing the relative visual (SI_firm,_ SI_foam_), somatosensory (SI_EO_, SI_EC_) and vestibular contributions (SI_V_) to balance (Table 2)[24]. An SI ≤ 0 indicates a destabilization of postural control. The advantage of using SIs is that they minimize the inherent intra- and inter-individual variability in MTV COP measures[18].

**Table 2:** Stability Index Formula and Description.

Stability Indices	Determines the relative contribution of the…
**SI_firm_** = 1—Log(FirmEO + 1)/Log(FirmEC + 1)	Visual system on a firm surface (baseline)
**SI_foam_** = 1—Log(FoamEO + 1)/Log(FoamEC + 1)	Visual system on an unstable or Foam surface, i.e. the somatosensory system is disrupted
**SI_EO_** = 1—Log(FirmEO + 1)/Log(FoamEO + 1)	Somatosensory system with Eyes Open.
**SI_EC_** = 1—Log(FirmEC + 1)/Log(FoamEC + 1)	Somatosensory system with Eyes Closed, i.e. vision is disrupted
**SI_V_** = 1—Log(FirmEO + 1)/Log(FoamEC + 1)	Vestibular system while Vision and Somatosensory systems are disrupted (most challenging)

Note: Under ideal conditions, all sensory systems work in concert therefore some redundancy exists. COP in conditions of increasing difficulty are measured. To determine the Stability Index, the visual and/or somatosensory systems are experimentally disrupted to derive the relative contribution of each system, however, the vestibular system is never experimentally disrupted. This protocol has been validated and has been used consistently for clinical assessment.

### Secondary Outcomes

#### Timed One-Leg Stance (OLS)

The OLS protocol requires the subject to raise one foot off the ground[70]. Time up to 30 seconds is recorded. They are instructed to keep the arms down at their sides and any touch of foot or hand to a surface stops the timer. The task is performed three times with eyes open and the longest duration of the three trials is used in the analysis. The ability to stand on a single-leg is an important predictor of falls in the elderly[70].

#### Physical Function

The “Chair sit-and-reach test” (CSRT) was used to assess lower body flexibility, which is important for good posture, normal gait patterns and various mobility tasks[71]. We measured the number of inches (+ or-) between extended fingers and the tip of the toe, as the participant extended the legs and reached their hands toward the toes, in a sitting position in front of a chair[71,72]. The “30-Second Chair Stand*”* 30s-CS was used to assess lower body strength, needed for numerous tasks such as climbing stairs, walking, and getting out of a chair, tub or car[73]. We measured the number of full stands that can be completed in 30 seconds with arms folded across the chest[71].

### Instrumentation

Custom software was developed to interface wirelessly with the WBB in order to collect COP data[27,74]. Data collection software was developed in C# using Microsoft Visual Studio and the.NET framework. WBB calibration provides an accurate measure of COP[27,75]. The data were sampled at 100 Hz and filtered using a Butterworth filter with a low pass cut-off frequency of 10 Hz[76]. COP data extraction software was written in MATLAB (MathWorks, Inc., MA) in order to calculate MTV. The WBB derives COP data sent by 4 force plate sensors on the platform (Fig 2B). Changes in COP (mm/s), sampled over 30s, are derived from the stream of sensor data sent to a laptop via Bluetooth. The WBB in a normal population was found to have a test-retest reliability of 0.66–0.94 and a between-device reliability of 0.77–0.89 when compared to a laboratory-grade, computerized force plate[74]. Furthermore, the test-retest reliability of the WBB was found to be reliable among a VI population with ICCs ranging from 0.88 to 0.94 and sensitive to modifications in sensory inputs[27]. In Jeter et. al.[27], the reliability for the Foam EC condition was equivalent to the Firm EC condition (both with ICC equal to 0.88), suggesting that while COP displacement may be relatively larger due to the more challenging Foam condition, the reliability is the same.

For the purposes of this study, the same device was used with our small number of participants. Bartlett et al. has shown that COP measures on the WBB do not deteriorate over time after heavy use, especially when using the same device across time points[77]. Given the infrequent use of the device in our study, it is reasonable to assume that the WBB was consistent throughout this study.

### Statistical Analysis

#### Primary Outcomes

All statistical analyses were conducted using IBM SPSS Version 22.0. The assumption of normally distributed data was verified using the Kolmogorov-Smirnov Test on the difference scores (post intervention minus baseline measures) in the primary dependent variable (absolute COP) for each group. The difference scores for all absolute COP variables met the condition of a normal distribution (all p > 0.05). Therefore, parametric statistical tests were used to analyze the primary outcomes. Independent t-tests were conducted to determine baseline differences between groups. This study was not powered to detect between group differences following the AYT, so significance of pre-post changes was assessed by paired samples t-tests within each group for all outcomes. Within-group confidence intervals were derived from the paired samples t-tests. Effect sizes for the primary outcomes were calculated as the partial eta squared, η^2^ = *t* ^2^/(*t* ^2^ + (N-1)). We use the Ferguson (2009)[78] effect size criterion for η^2^: small = 0.2, moderate = 0.5, large = 0.8. All data was coded to mask group assignment.

#### Secondary Outcomes

The assumption of normally distributed data was marginal for 2 of 3 of the secondary outcomes (OLS, p = 0.06 and 30s Chair Stand, p = 0.07) and was violated for one (CSRT, p = 0.001). We opted to evaluate all three secondary outcomes with the non-parametric version of the paired t-test, the Wilcoxon Signed Rank Test. The effect size was calculated by dividing the *z* score by the square root of N (*r* = *z*/√N). In this case, N = the number of observations over each time point, not the number of subjects. Ferguson's (2009)[78] effect size criterion for *r* is the following: small = 0.04, moderate = 0.25, large = 0.64.

## Results

### Subjects’ Characteristics and Study Completion

Twenty-nine participants were screened for eligibility (Fig 1). Twenty-one legally blind participants met the inclusion criteria and were randomized to the 8-week AYT program (n = 11, mean (SD) age = 55(17); 3 Males) or waitlist control (n = 10, mean (SD) age = 55 (10), 3 Males). Demographic information and visual history are described in Table 3. As an RCT, age differences at baseline were not a factor (t(15) = 0.014, p = 0.99). However, we conducted an additional analysis to determine if postural stability differed between age groups, as follows. Our sample is comprised of participants in age ranging from 27–85 (median age = 59). If we re-characterize our sample regardless of original group assignment (i.e. AYT or waitlist) into two new groups separated using the median age of 59 as a cut-off (subjects < 60 years old, n = 9, and subjects ≥60 years old, n = 8), we can conduct an independent samples t-test between groups on the baseline value for the most difficult condition where we might see the most differences in postural stability, Foam EC. We found no significant difference between age groups (t(15) = -1.43, p = 0.17), suggesting the groups were equivalent at baseline regardless of age or postural stability.

**Table 3:** Demographic and Visual History.

Group Assignment	Age	Gender	Race	Diagnosis	OS	OD	Visual Field
Yoga	44	M	Caucasian	Retinitis Pigmentosa	20/30	20/30	<10
Yoga	64	F	Caucasian	Congenital glaucoma, aphakia, prior retinal detachment	CF 2'	BLP	
Yoga	54	M	African American	Diabetic Retinopathy	LP	LP	
Yoga	65	M	African American	Childhood accident, firecracker	BLP	BLP	
Yoga	85	F	Caucasian	AMD	CF @ 3'	HM	
Yoga	38	F	Caucasian	Congenital cataracts, aphakia with band keratopathy, phthisis OD	CF @ 1'	NLP	
Yoga	62	F	Caucasian	Retinopathy of Prematurity	NLP	BLP	
Yoga	27	F	African American	Stargardt's Disease	20/320	20/320	
Yoga	58	F	Caucasian	Retinitis Pigmentosa	20/70	20/70	<10
Waitlist	60	F	Caucasian	Retinitis Pigmentosa	CF	HM	<10
Waitlist	64	F	African American	Stargardt's Maculopathy	20/320	20/320	
Waitlist	60	M	Caucasian	Retinitis Pigmentosa	BLP	BLP	
Waitlist	60	F	Caucasian	Nystagmus, Congenital Cataracts, Band Keratopathy	HM	NLP	
Waitlist	33	F	Hispanic	Optic Nerve Damage	LP	LP	
Waitlist	49	M	Caucasian	Stargardt's Disease	20/300	20/300	
Waitlist	56	M	Caucasian	Ischemic Optic Neuropathy	LP	LP	
Waitlist	59	F	Caucasian	End-stage Glaucoma	LP	LP	

OS, Left Eye; OD, Right Eye; CF, Counting Fingers; HM, Hand Motion; LP, Light Perception; BLP, Bare Light Perception; NLP, No Light Perception

Two participants in the AYT group did not complete the study. One participant opted out after two yoga classes due to dissatisfaction with the yoga program and expressed a preference for private yoga lessons. Another participant developed pain in her legs due to pre-existing peripheral neuropathy that was not reported during screening, which was an exclusion criterion. Two participants in the waitlist group were excluded from the final analysis; one due to newly developed health issues unrelated to the program and another due to time constraints and health prohibiting evaluation at study completion during the post-AYT assessment period. The dropout rate was 19% and no study related adverse health events were reported. Subjects completing the intervention participated in an average of 6.6 (SD 0.73) out of 8 total classes, or 82% overall. According to the practice logs, participants reported practicing at least twice a week at home 90% of the time.

### Primary Outcomes

#### Mean Total Velocity (MTV) COP

There were no significant differences at baseline between groups for all COP variables (all p > 0.05). Absolute values of MTV COP significantly increased from baseline to post-AYT in the Foam_EO_ and Foam_EC_ conditions (t(8) = -3.66, p = 0.01 and t(8) = -3.90, p = 0.01, respectively (See Table 4). Correspondingly, effect size calculations revealed a moderate effect of intervention for these two conditions (Table 4). No statistically significant changes in the AYT group were observed pre-post in the Firm_EO_ or Firm_EC_ conditions (p > 0.05). No statistically significant pre-post changes were observed in the waitlist control group (all p > 0.05) for any MTV COP variables.

**Table 4:** Primary Outcomes—Absolute COP MTV (mm/s) and Relative Stability Indices.

		Group Mean(SD)				
Group	Outcome	Baseline	Post	95% CI of the Mean Difference	P-value	Effect Size (η^2^)
*AYT*	Firm_EO_	11.91 (3.14)	12.02 (2.21)	-0.10 (-2.39 2.18)	0.92	0.00
	Firm_EC_	12.65 (2.62)	13.04 (2.50)	-0.39 (-2.35 1.58)	0.66	0.03
	Foam_EO_	22.62 (6.12)	29.03 (7.49)	-6.41 (-10.45–2.37)	0.01*	0.63
	Foam_EC_	22.90 (3.19)	33.09 (10.03)	-10.19 (-16.22–4.16)	0.01*	0.66
*Waitlist*	Firm_EO_	14.07 (5.01)	14.28 (4.38)	-0.21 (-2.26 1.84)	0.82	0.01
	Firm_EC_	13.49 (4.93)	14.49 (4.14)	-1.00 (-2.86 0.86)	0.24	0.19
	Foam_EO_	23.82 (8.25)	25.83 (6.70)	-2.00 (-6.82 2.80)	0.36	0.12
	Foam_EC_	27.80 (7.30)	31.08 (8.29)	-3.29 (-8.98 2.40)	0.21	0.21
*AYT*	SI_Firm_	0.02 (0.08)	0.03 (0.04)	-0.01 (-0.07 0.06)	0.82	0.01
	SI_Foam_	0.01 (0.08)	0.03 (0.04)	-0.02 (-0.07 0.03)	0.34	0.11
	SI_EO_	0.19 (0.06)	0.24 (0.07)	-0.05 (-0.10–0.01)	0.04*	0.42
	SI_EC_	0.18 (0.07)	0.25 (0.05)	-0.07 (-0.11–0.03)	0.01*	0.66
	SI_V_	0.20 (0.08)	0.26 (0.06)	-0.07 (-0.13–0.01)	0.04*	0.43
*Waitlist*	SI_Firm_	-0.02 (0.03)	0.01 (0.05)	-0.02 (-0.06 0.02)	0.24	0.19
	SI_Foam_	0.05 (0.08)	0.05 (0.05)	0.00 (-0.08 0.08)	1.00	0.00
	SI_EO_	0.16 (0.03)	0.17 (0.08)	-0.16 (-0.08 0.05)	0.58	0.05
	SI_EC_	0.21 (0.06)	0.21 (0.06)	0.01 (-0.07 0.07)	0.97	0.00
	SI_V_	0.20 (0.06)	0.22 (0.06)	-0.02 (-0.08 0.04)	0.48	0.07

Primary outcomes are analyzed by paired samples t-tests. 95% CI are confidence intervals for the mean difference pre-post t-tests. Effect sizes for primary outcomes were calculated as the partial eta squared, η^2^ = t^2^/(t^2^ + (N-1)). We use the Ferguson (2009) effect size criteria for η^2^: small = 0.2, moderate = 0.5, large = 0.8. SI = Stability Index, EO = Eyes Open, EC = Eyes Closed, V = Vestibular,

* = Statistically significant.

#### Stability Index (SI)

The relative contribution of sensory input measured by the stability index (SI) is displayed in Table 4. A significant baseline—post-AYT increase in the somatosensory contribution to balance was found in the AYT group in the SI_EO_ (t(8) = -2.42, p = 0.04) and the SI_EC_ conditions (t(8) = -3.96, p = 0.01; Table 4). A significant pre-post increase in SI_V_ was observed (t(8) = -2.47, p = 0.04) in the AYT group, indicating an increase in vestibular contribution to balance. In contrast, there were no statistically significant changes observed in the control group (all p > 0.05). As expected, reduced visual contribution or visual dependency to balance (i.e., SI_Firm,_ SI_Foam)_ was present in VI and no significant pre-post changes were found for either group (all p > 0.05).

### Secondary Outcomes

A Wilcoxon Signed Rank Test revealed a statistically significant increase in the OLS-EO (*z* = -2.10, p = 0.04), the Chair Sit and Reach test (*z* = -2.22, p = 0.01) suggesting an increase in flexibility, and the 30s Chair Stand (*z* = -1.98, p = 0.05) suggesting an increase in lower body strength, following the AYT program (Table 5). Effect sizes were in the moderate to large range (i.e., *r* = 0.47–0.60). No statistically significant effects were observed for OLS or fitness measures in the waitlist control group (all p > 0.05).

**Table 5:** Secondary Outcomes.

		Median (IQR)			
Group	Outcomes	Baseline	Post	P-value	Effect Size (*r*)
*AYT*	OLS-EO (s)	4.46 (12.94)	6.87 (26.25)	0.04*	0.49
	Chair Sit & Reach (in)	0.00 (4.00)	1.50 (4.25)	0.01*	0.60
	30s Chair Stand (s)	13.00 (4.50)	14.00 (5.50)	0.05*	0.47
*Waitlist*	OLS-EO (s)	4.93 (7.04)	7.15 (6.45)	0.31	0.25
	Chair Sit & Reach (in)	-2.5 (8.5)	-3.25 (7.63)	0.75	0.08
	30s Chair Stand (s)	13.00 (4.50)	11.50 (6)	0.31	0.26

Secondary outcomes were calculated using the Wilcoxon Signed Rank test and effect sizes were calculated as r = z/√N, where N is the number of observations over the two time points. Ferguson's (2009) effect size for r is the following criteria: small = 0.04, moderate = 0.25, large = 0.64. IQR = Interquartile Range, (in) = Inches, (s) Seconds,

* = Significant Effect, OLS = One-Legged Stand, EO = Eyes Open.

### Sample Size Calculation for Future Studies

In order to develop the intervention protocol and plan for larger scale RCTs, we can estimate the sample size based on these results for future studies. In a future study, investigators may be interested in using an Analysis of Covariance (ANCOVA) to determine post-intervention effects between-groups, while controlling for baseline or some other covariate of interest (e.g., age). Between-group comparisons for the post-intervention measures control for non-specific effects (e.g., social interactions, placebo) that are difficult to manage in within-group comparisons. Here, we estimate an effect size based on the mean difference between the post-AYT measures in the absolute COP Foam EC condition divided by the pooled standard deviation. In an analysis of covariance (with baseline as the covariate) to detect an effect size of 0.45 for the post-intervention difference between treatment and control, participation by 41 participants achieves 80% power at the 0.05 alpha level. Anticipating a 20% dropout rate, one may enroll 50 participants total.

## Discussion

The goal of this study was to determine the effect of AYT on COP and on the sensory contributions to postural stability in VI participants. Sensory integration of movement information from visual, somatosensory and vestibular systems contributes to postural stability[22]. After vision loss, re-weighting of the remaining sensory inputs is important for generating new postural strategies to maintain upright balance[15,41]. Following the 8-week AYT program, this study found a significant increase in the MTV for the Foam_EO_ and Foam_EC_ conditions in which both somatosensory and visual systems were disrupted. Correspondingly, the relative contributions of the somatosensory (SI_EO_, SI_EC_) and vestibular systems (SI_V_) in our treatment group increased. As hypothesized for this VI population, vision did not play a significant role in postural stability, as indicated by non-significant changes in either MTV or SIs. Moreover, the waitlist control group showed no significant changes in any outcomes. Additionally, we determined that differences in the age range in our study population were not a factor in the results observed.

To our knowledge, this is the first time sensory input to postural stability has been evaluated as an objective outcome before and after yoga training in an aging, VI population. In an earlier review of yoga for balance in a healthy population[79], only one of 15 studies measured COP in a dynamic standing task, which suggests that measures of postural stability are underutilized. COP measures are objective and may be more sensitive to changes in postural stability in a clinical population, and less influenced by placebo effects, than self-reported outcome measures[79]. Despite the small sample size in the current study, the results showed a strong effect in the AYT group but not the control group, consistent across variables. In addition, the novel use of the WBB as a standalone posturography device introduces a possible cost-effective, portable alternative for measuring postural stability. Using COP and the mCTSIB protocol, the contribution of each sensory input in a clinical population can be evaluated, in order to devise tailored rehabilitation programs in the future.

**Fig 3:**
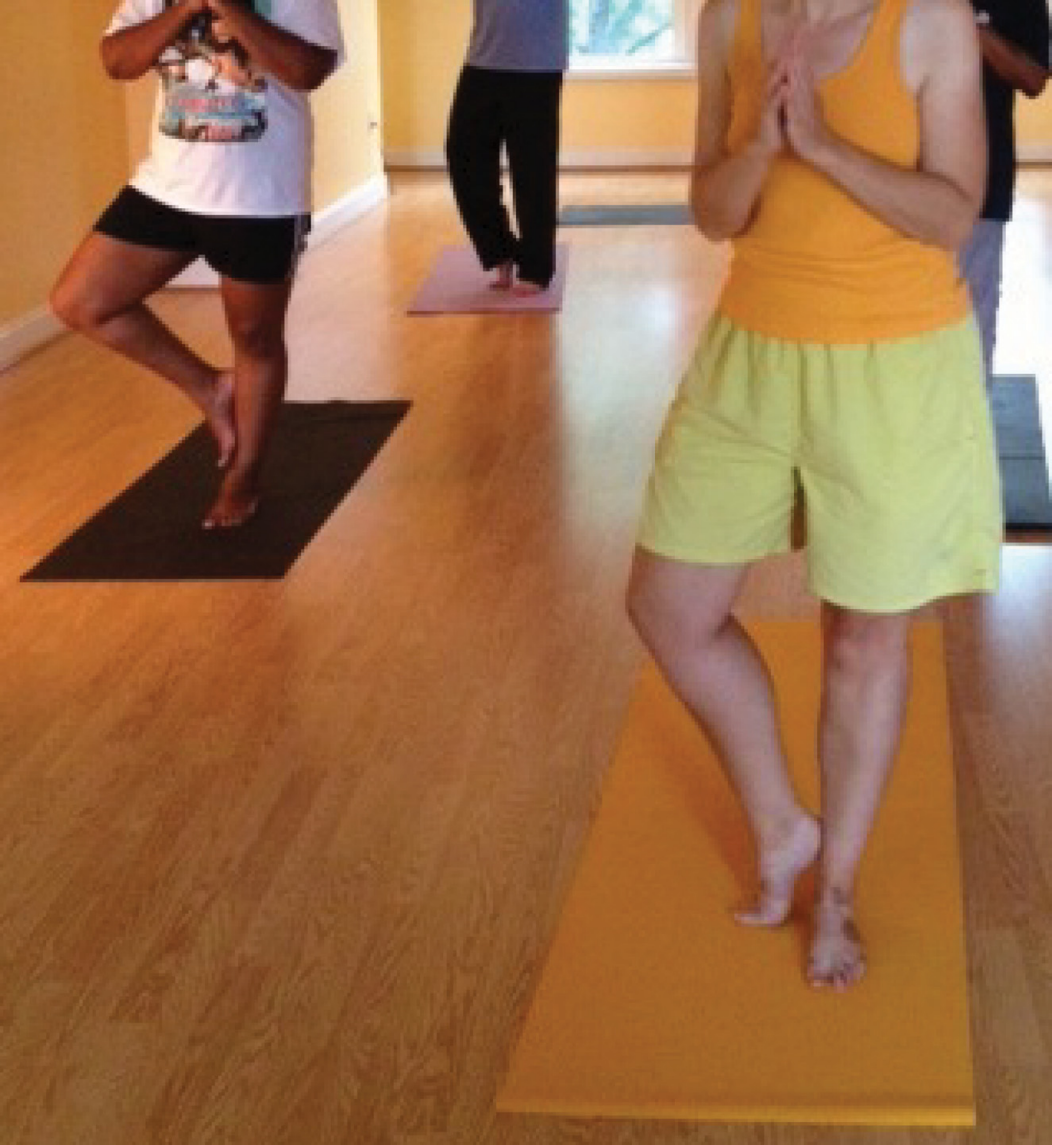
Visually impaired students performing tree pose.

The results of the present study support posture COP stabilization rather than a minimization hypothesis, which strongly suggests that AYT training could engage self-correcting mechanisms for postural control, such as up-weighting proprioceptive feedback, as well as increasing lower body strength and flexibility, which can potentially reduce fall risk. COP stabilization suggests that the greater COP displacement observed in this study represents greater integration of the remaining sensory systems to activate appropriate muscle responses used to control postural stability. This conclusion is further strengthened by the significant improvements in flexibility observed with the CSRT, consistent with the findings of Wayne et al.[52]. Increased lower body flexibility is important for normal gait patterns during walking. The increase in lower body strength suggested by the 30s Chair Stand and OLS results further supports this claim. While the OLS does not provide information about sensory inputs, the ability to stand on a single leg is an important predictor of falls[70]. In addition, the biomechanical demands of certain poses, such as tree pose (Fig 3), have been shown to match balance potential[80].

Furthermore and perhaps more compelling evidence in support of the stabilization hypothesis comes from a comparison of the post-AYT SI measures from our VI group to baseline SIs collected from normally-sighted, young adults (See S1 Appendix). The data set for the normally-sighted individuals has been published elsewhere[27] as part of a separate study to establish reliability of the WBB and therefore, we calculated the SI from the normal data for the purp ose of comparison with the SI for VI in the current paper. Comparing baseline SI from normally sighted individuals to post-AYT VI using independent samples t-tests indicate no difference between groups (SI_EC_: t(28) = 0.51, p = 0.62, SI_VE_: t(28) = -1.37, p = 0.18), suggesting that after AYT training the VI group approached the normally-sighted baseline (Figure A in S1 Appendix). These findings are consistent with those of Tsang et. al.[49] who found, in a cross-sectional study, that the vestibular contribution achieved by elderly Tai Chi practitioners was similar to that of young adult subjects rather than that of elderly non-Tai Chi practitioners, suggesting that despite age differences sensory re-weighting is amenable to training. We did find a significant difference between normal and VI for SI_EO_; however, this is to be expected as the normally-sighted group had full use of visual sensory information in EO conditions. The somatosensory contribution in the VI group is significantly greater than in the normal group, suggesting a greater use of somatosensory information. Although absolute COP values are greater than for healthy, normals in cross-sectional analyses, as evidenced by several notable studies supporting the COP minimization hypothesis[16,24,26], COP measures may in fact reflect different postural strategies depending on the population[47,48]. The relative contributions of sensory systems after training may reflect these differences more clearly.

Wolf and colleagues[51] administered a 15-week Tai Chi program to healthy elderly adults (> 70 years of age) and found an increase in COP in somatosensory conditions with both EO and EC. This was reported as a negative effect compared to a group receiving computerized balance training. It was noted that the Tai Chi group reduced fear of falling outcomes while the balance group did not. Wolf et al.[51] raised the possibility that training resulting in greater COP could lead to improved strategies for maintaining a wider base of support and improved confidence. Tai Chi practice, similar to yoga, involves shifting one’s weight between double and single stance, body rotation and awareness of alignment[81], such that maintaining balance displacement within the limits of stability and/or base of support is constantly challenged[49]. More recently, Wayne et. al.[52] compared a group of Tai Chi experts (average age 63 years, with 25 years of experience) to a group of Tai Chi naïve participants (average age 65) in a cross-sectional study of community dwelling healthy adults and reported a trend towards higher MTV in the expert group. The naïve participants were then randomized to usual care or 6-months of Tai Chi. After training in Tai Chi, the naïve participants developed increased MTV and had values similar to the Tai Chi experts, which is in concordance with the findings in VI adults after AYT reported here. Gyllensten found that Tai Chi practitioners had better stability limits and body awareness compared to younger control subjects[82]. Increased limits of stability and engagement in controlled movements with improved balance control were reported in a group of Parkinson’s patients after Tai Chi[50], again supporting the plausibility of increased COP as a balance strategy for certain populations. Greater COP displacement has been has been reported to provide proprioceptive feedback in an elderly population[48], specifically feedback from the ankle joint, which could compensate for visual deficits. Furthermore, maintaining balance by sensory reweighting of somatosensory or vestibular systems may involve better use of hip or ankle postural control strategies[35].

The AYT was used to engage unimpaired sensory systems, i.e. somatosensory and vestibular, in order to compensate for vision loss in a safe environment that may have the added benefit of staving off conditions associated with age-related decline (e.g., deconditioning of motor and perceptual processes). Notably, it has been suggested that programs emphasizing multisensory training to improve balance may be more effective than programs emphasizing single sensory or modality training[31,36,37]. The practice of yoga may improve the quality of the sensory information for VI persons by targeted engagement of compensatory systems. Yoga, in general, may be desirable choice for the aging VI populations because classes are widely available, movements are low-impact, and poses can be modified to suit the individual although it would be desirable to train more yoga instructors on adaptations necessary to work with VI individuals. Yoga has also been shown to have a low rate of side effects, low risk of injury and no known interactions with prescription medications[83]. Finally, a review of 12 studies (8 RCTs) that directly compared yoga to exercise found that yoga was equal to or significantly better as judged by health-related outcomes such as improved quality of life and reduced stress, in both healthy and clinical populations[84].

### Limitations

We used COP as a surrogate marker for falls. Without a prospective study measuring falls as an outcome, it is difficult to draw any conclusions about reduced fall risk. In the future, a prospective study with a longer-term yoga program and follow-up is warranted. Measuring static posturography in this study has granted us an understanding for multisensory integration and training unimpaired systems, however, measuring dynamic posturography in the future may be more relevant to determining anticipatory (e.g., obstacle avoidance) or compensatory (e.g., stumble recovery) postural adjustments after unpredictable perturbations during walking. Falls are a result of several intrinsic and extrinsic risk factors; and it will require a multifactorial approach to identify and manage those risks. Furthermore, in this study somatosensory and vestibular contributions to balance were inferred from COP data. In the future, it would be important to directly measure these contributions.

Limits of stability (LOS) were not measured in this study. LOS can be defined as when the center of gravity falls outside the base of support leading to an increase risk of falls[67] and is usually measured using the Functional Reach Test[85]. COP stabilization may imply an increase in the LOS. Alternatively, it is also possible that LOS could increase with COP minimization when different conditions are met the by the population of interest. Whether LOS is correlated with changes in COP is an empirical question. At least one study has claimed that there was a low correlation (r = 0.38) between the functional reach test and displacement of center of pressure in healthy, elderly people ages 60–85[86].

Unlike a lab grade force platform, the Wii balance board only measures force in the vertical direction and is not able to detect the contribution of shear forces. Several studies have demonstrated that the Wii balance board has excellent reliability and validity when compared to a force plate[74], including our own reliability study in a visually impaired and normal population[27]. We don’t expect that the displacement in our study would exceed the displacement one might expect in a dynamic task or single leg stand. We expect the effect of the shear forces on the COP to be minimal in the double-leg quiet stance. There is also evidence that the shear measurements may not be reliable in studies of postural responses among aging individuals[67,87]. Since the WBB was not designed to measure shear, we are not able to account for it in this study however, there is research supporting vertical COP as sufficient to detect balance impairments among a diverse population[43].

Despite specific criteria to minimize heterogeneity in our VI population, there are likely to be individual differences. For example, visual function can vary according to severity, duration, and type of disease and may limit the interpretation of results, as we were unable to perform subgroup analyses given the small sample size in the current study. There is evidence that vision loss may accelerate the effects of postural instability during a wide age range[5] and considering the pilot nature of this study, we did not exclude participants based on age. However, the heterogeneity of VI participants in our trial may more accurately represent clinical situations, reflecting generalizability. The clinical significance of these results may not be immediately apparent without a long-term prospective study to measure falls and sustainability of effects. Qualitative data that was collected in the current study, to be presented elsewhere, may be more informative about potential clinically relevant outcomes. Due to the small sample size the present trial was not sufficiently powered to detect changes between the two groups for the outcomes after AYT; therefore a larger scale trial is warranted to explore those differences. Sampling bias occurs when there are systematic differences between groups being compared. In order to minimize the possibility of bias, we randomized the group assignment and further checked for statistical differences at baseline, of which we found none. Since this was an exploratory study, we did not correct for multiple statistical comparisons for the several tests included.

## Conclusions

We conclude that greater MTV COP displacement observed post-AYT supports a COP stabilization hypothesis in a VI population. AYT training may activate better use of the remaining somatosensory and vestibular inputs to postural stability. This may be a meaningful and necessary self-correcting strategy for postural stability for a VI population at risk for falls. Knowing the relative sensory inputs to postural stability may help us understand the contribution of each system under different conditions. Multisensory training of the visual, vestibular or somatosensory systems has shown to significantly improve stability[31,36]. Therefore, it may prove beneficial to examine sensory contributions to balance in future studies, as it may reduce fall risk and improve postural stability.

## Supporting Information

S1 Protocol IRB protocol approved for the trial. (DOC)

S1 CONSORT Checklist CONSORT Checklist. (DOC)

S1 Appendix Normally-Sighted versus Visually Impaired Stability Indices. (DOCX)

S1 Dataset Primary Dataset for Absolute Center of Pressure, Mean Total Velocity (mm/s) in 4 Conditions. (XLSX)

S2 Dataset Secondary Dataset for OLS, Chair Sit and Reach and 30s Chair Stand. (XLSX)
