# Supplementary material for: Ashtanga-Based Yoga Therapy Increases the Sensory Contribution to Postural Stability in Visually-Impaired Persons at Risk for Falls as Measured by the Wii Balance Board: A Pilot Randomized Controlled Trial
Source: PLoS One. 2015 Jun 24;10(6):e0129646. doi: 10.1371/journal.pone.0129646 (PMC4479589; doi:10.1371/journal.pone.0129646)
Supplement: S1 Protocol — (DOC) [file pone.0129646.s001.doc]

**Johns Hopkins Medicine - eForm A**

- **Use the section headings to write the eForm A, inserting the appropriate material in each. If a section is not applicable, leave heading in and insert N/A.**
- **When submitting eForm A (new or revised), enter the date submitted to the field at the top of eForm A.**

***************************************************************************************************

1. **Abstract**
2. Provide no more than a one page research abstract briefly stating the problem, the research hypothesis, and the importance of the research.

Patients with retinal degenerative diseases experience a slowly progressive, unpredictable loss of vision which eventually leads to bare or no light perception, posing a continuous threat to patients’ independence. It is hypothesized that sleep disturbances in individuals with severe vision loss may be related to the extent of photoreceptor loss and narrowing field of view leading to reductions in retinally mediated light. Light regulates the circadian cycle in humans and when it is disrupted by the loss of photoreceptor and/or photosensitive cells, problems related to sleep might occur. We propose to examine differences in negative psychosocial indicators in order to determine factors related to sleep distress.

Loss of vision is likewise accompanied by poor balance, postural instability, increased risk of falls and decreased mobility, significantly reducing independence and quality of life in visually impaired (VI) patients. For persons with severe visual impairment, the risk of falls doubles. Adults may also develop “fear of falling” (FOF) and may actually be a more widespread problem that further limits independence, reduces activity level and confidence. Postural sway becomes more pronounced if kinesthetic feedback is perturbed. Similarly, a VI individual may be at greater risk of falling when walking on thick carpet with thick-soled shoes, or on a sandy beach. Therefore, it is important to determine if postural instability or balance deficits exist among the VI population. Static imbalance and compensatory sensory inputs can be reliably determined by measuring postural instability, which is typically done using a computerized force platform. Such platforms are expensive (~$10K-$60K) and rarely available for use by outside researchers. Measuring static balance may be an important surrogate for falls[^8^](#_ENREF_8). Therefore, we introduce the use of the WBB as an objective measures for static balance which has never been used in the VI population.

Learning to access other non-visual information, such as improving proprioceptive or kinesthetic awareness, is an important factor to consider in severe vision loss. Yoga (e.g. postures, meditation, breathing) has been shown to be a viable intervention to improve sleep disturbances, mood disturbances, and balance, in healthy individuals and those with other chronic diseases. In this pilot study, we aim to evaluate and implement a yoga treatment specifically tailored to the VI population for its feasibility to alleviate sleep disturbances, psychosocial indicators, fear of falling and balance. Measuring participant experience through anonymous exit surveys will further help evaluate the yoga intervention. These data are especially important since there have been no previous publications from other researchers (with the exception of our first paper[^9^](#_ENREF_9)) involving yoga and interventions in the VI population. Our future research goals include the conduct of clinical trials involving mind-body interventions in visually impaired patients, aiming to decrease secondary symptoms and improve quality of life.

1. **Objectives** (include all primary and secondary objectives)

1) To explore the relationship between psychophysical visual function in patients with severe vision loss and sleep disturbances, negative psychosocial factors and balance.

2) To test the feasibility of a yoga intervention for reducing secondary symptoms.

3) To determine the use of a commercially available Nintendo Wii Balance Board^TM^ (WBB) to measure static balance in visually impaired individuals.

1. **Background** (briefly describe pre-clinical and clinical data, current experience with procedures, drug or device, and any other relevant information to justify the research)

**Yoga as an approach.** Yoga has been shown to be a viable intervention for improved sleep disturbances, mood disturbances, fear of falling (FOF), and balance, in healthy individuals or those with other chronic diseases. Yoga is an integrated system of postures, movement, breath and meditation and is easily implemented regardless of age or level of experience. The Ashtanga-based Yoga Therapy (AYT) will be evaluated for its feasibility. The main component of the AYT is movement with the breath. The breathing involved, called *ujjayi* breathing, is a specialized technique that emphasizes use of the diaphragm. Poor breathing patterns can lead to anxiety and chronic overstimulation of the sympathetic nervous system in emphysema patients. On the other hand, normal, healthy breathing can influence cardiovascular factors producing a stabilizing effect. Teaching yoga to low vision patients requires simple modifications to postures, clear descriptions, and hands-on adjustments.

**Sleep disturbances and psychophysical measures***.* Very little is understood about the relationship between psychophysical measures (visual field, contrast sensitivity, acuity) and sleep disturbance as VI progresses. Measuring relative rod and cone sensitivities with a two-filter dark-adapted ganzfeld test system will determine the extent of retinal degeneration.

**Yoga and Psychosocial Factors***.* The impact of visual loss on negative mood states (e.g. anxiety,

depression) may be an independent factor contributing to disturbed sleep in individuals with low vision. Patients have reported transient episodes of vision loss or reductions in visual fields that are attributed to stress and fatigue. It has been argued that positive self-efficacy governs the ability to remain resilient to adversity, such as adapting to progressive vision loss, thus reducing susceptibility to depression and negative psychosocial states arising from stress. Positive results after yoga have been reported for stress, depression and anxiety that may further alleviate disturbed sleep. It’s possible that sleep disturbances are not strictly a result of retinal degeneration; therefore, including validated measures that evaluate stress, anxiety, depression and related factors (e.g. self-efficacy) will help distinguish the relative contributions of negative psychosocial factors.

**Vision Loss, Falls, and Fear of Falling.**

Vision plays a dominant role in a person’s ability to navigate through the environment. The reduced ability to respond to visual cues for individuals with VI leads to increased barriers to independence and risk of falling due to impaired balance. Balance is critical as a prerequisite to movement and impaired balance is considered an important predictor of falls[^8^](#_ENREF_8). Posture results from a proper integration of movement between the head, torso and limbs in order to maintain balance. Vestibular (e.g., changes in head position) and somatosensory systems (e.g. proprioception) must work in concert with vision in order to maintain equilibrium. When one dimension is compromised as it is in severe vision loss, alternative strategies targeting the remaining dimensions that contribute to balance must be considered. Adults may also develop a “fear of falling” (FOF) and may actually be a more widespread problem that further limits independence and participation in activities, and reduces confidence.

Balance and Postural Stability: There are three distinct yet integrated sensory input streams that regulate postural stability: kinesthetic (e.g. proprioception), vestibular (e.g. changes in head position), and visual system (e.g. visual fields)[^5-7^](#_ENREF_5). When vision is impaired, balance is also impaired[^7^](#_ENREF_7). For example, in adults with normal vision standing with their eyes closed an increase postural sway by 20-70% was reported[^10^](#_ENREF_10). Individuals with visual impairment (VI) – i.e., vision loss that cannot be corrected optically, surgically, or medically – have irreparable damage to one of the input streams contributing to balance and postural stability. *Static balance* is defined as the ability to maintain the center of gravity within a base of support in a quiet upright position during standing or sitting. As vision loss progresses, there is further decrease in postural stability. Impaired balance is considered a predictor of falls and fear of falling (FOF)[^11^](#_ENREF_11).

The WBB is a commercially available portable force plate device that is part of the Nintendo Wii video gaming system. It was recently validated for its ability to quantify center of pressure (COP)[^12^](#_ENREF_12) an indicator of postural instability. The WBB force plate data is accessible by a laptop equipped with Bluetooth and open source software. It has been tested for validity with a normal elderly population, and was found to have a test-retest reliability of 0.66-0.94 and a between-device reliability of 0.77-0.89 when compared to a research-grade computerized force plate[^12^](#_ENREF_12).

**Yoga and balance.** As vision declines, adopting training strategies that promote the use of other sensory/vestibular information may aid in the development of awareness of the body as it moves in space. Therefore, cultivating the integration of mind and body through yoga may yield favorable outcomes as a vehicle to improve balance. Using yoga as an appropriate proprioceptive strategy for the elderly has proven more beneficial when compared to a matched group participating in other physical activities. Yoga improved hip extension, increased stride length, thus improving gait in healthy elders. Balance improved substantially in a separate study using a timed, one-legged balance test after yoga training in healthy adults. Older adults who participated in a yoga program had reduced psychological stress and anxiety inherent in “fear of falling”[^13^](#_ENREF_13). Yoga has been shown to be an effective intervention for falls, balance and FOF in normally sighted population[^14^](#_ENREF_14) however; it is yet to be evaluated for these intentions in the blind population.

**AYT Validation.** The purpose of yoga is to integrate mind and body and therefore incorporates a contemplative or mindfulness component that sets it apart from regular exercise. ‘Mindfulness’ in this setting promotes self-awareness in a non-competitive, non-judgmental environment and at the same time promotes awareness to muscular movements, alignment and the breath. Study patients are more likely to comply with an intervention if it is safe, engaging and easy to follow. Therefore, measuring compliance with practice logs and participant experience through exit surveys will further help evaluate the yoga intervention. Finally, evaluating the fidelity of treatment or how accurately the yoga protocol is reproduced will be a step forward in the effective standardization of the AYT as a means of improving quality of life in VI. We will videotape and/or photograph all yoga sessions and randomly select 4-6 classes for scoring. Fidelity is based on how closely the instructor adhered to the yoga protocol (i.e. sequence of poses, duration) during the class, and will be scored according to checklist criteria, in order to provide feedback and additional training to the instructor.

In addition to assessing fidelity of treatment, these recordings and/or photographs may be shown at scientific and non-scientific professional meetings. Recordings will only be used for educational or scientific purposes with the participant’s consent.

1. **Study Procedures**
2. ***Study design, including the sequence and timing of study procedures***

***(distinguish research procedures from those that are part of routine care).***

This pilot study will determine the feasibility of the Ashtanga-based Yoga Therapy (YT) in VI subjects participating in a small scale randomized controlled trial. Each session will take approximately 1-3 hours over the course of 12 weeks.

**General Overview:** Baseline measures will be collected prior to the intervention, and participants will be randomized into two groups. An 8-week RCT will be conducted with AYT administered to one group, while the other group is waitlisted; post-tests (all measures from baseline except vision) are collected from all subjects. Then the 8 weeks of AYT are administered to the waitlist group, followed by another set of post-tests. Having a waitlist control group will allow us to compare the effects of AYT on the test measures to that of usual (i.e., no) care, while the final set of post-tests will allow us to compare effect of AYT on the two groups, and determine retention of any gains from the first AYT block.

After explaining the purpose and methods of the study and obtaining informed consent (the consent form will be read to potential participants due to their visual impairment), visual acuity and contrast sensitivity will be evaluated, and background medical and ocular history information will be obtained (30 minutes).

We anticipate that all procedures will take place at the Johns Hopkins Wilmer Eye Institute’s Lions Vision Center. We propose to contact potential subjects by phone and/or email to determine whether they meet the entry criteria and are interested. Enrolled subjects will first be assessed for all baseline measures and then be randomized into two equally sized groups to either start the AYT intervention immediately or to a waitlist control group receiving usual care during the initial 8-week intervention period. Waitlist control subjects will also receive the AYT intervention following the completion of the first AYT intervention group. Participants will convene weekly during the study period and Dr. Jeter (or a trained instructor) will teach the AYT intervention since she has previous experience teaching yoga to visually impaired individuals. Dr. Jeter will also train volunteer yoga instructors in the yoga protocol. This has been done previously in our pilot study[^9^](#_ENREF_9). Participants will also be asked to practice the intervention at home twice a week in addition to the visit at the center using a CD with audio instructions created by Dr. Jeter. A Post-Assessment Battery (all measures from baseline) will be collected immediately following the intervention. Another set of post-tests will be administered to the waitlist group. The waitlist control group will allow us to compare the effects of AYT on the test measures to that of usual care, while the final set of post-tests will allow us to compare effect of AYT in the two groups, and determine retention of any gains from the first AYT block.

A baseline assessment battery for all variables of interest will be obtained during the baseline period. These measures include **visual function, sleep, psychosocial questionnaires**, **fear of falling (FOF), physical function** and **balance**. All baseline measures will be reassessed within two weeks after the AYT intervention has been completed.

**Visual Function**

Vision tests will be conducted to ascertain eligibility, including ETDRS visual acuity (VA), Pelli-Robson contrast sensitivity (CS) and Goldmann visual fields (VF), and background medical and ocular history information will be obtained.

*Dark-adapted full field flash testing:* This test is able to accommodate subjects with poor vision (including those with light perception only) by assessing dark-adapted sensitivities. The subject, using a two-alternative, forced-choice procedure, is required to detect single flashes presented at random intervals until a threshold is determined. This takes about 10 minutes per eye and is able to monitor changes in retinal disease. Detection ratios for long (red LED) and short (blue LED) wavelength sources can be used to estimate rod and cone contributions: rods are several orders of magnitude more sensitive to short wavelengths.

The dark-adapted full field flash testing described above will require the pharmacological numbing and dilation of the subjects’ pupils. In the case that a subject experiences a significant change in vision between visits, a dilated eye examination will be performed in order to determine the cause of the change, and appropriate referrals for further evaluation and/or treatment will be made as needed.

**Sleep Measures**

*Sleep Diaries.* During two weeks prior to the beginning of the treatment, subjects will complete a online *sleep diary* immediately upon waking to assess subjective impressions of sleep. Subjects are asked to estimate how long it took them to fall asleep, how many times they awoke, how long they were awake, and how much sleep was obtained. Subjects will complete visual analog scales (scores 0-10) assessing the perceived quality and degree of refreshment provided by their sleep. The diary has an evening section, which is completed before going to bed, which gathers data on vision, napping, use of analgesics, and use of centrally acting substances.

The *Pittsburgh Sleep Quality Index* (PSQI) questionnaire has been previously validated and is routinely used in various clinical settings and research. The 19-item PSQI questionnaire will be used to gauge sleep quality over the past month. It includes both qualitative and quantitative aspects of sleep, and evaluates seven subscale dimensions of sleep quality.

**Psychosocial Questionnaires**

We will administer six very brief online questionaires for various mood states. These questionnaires include the Profile of Mood States – Short Form (POMS-SF) [^15^](#_ENREF_15), Perceived Stress Scale (PSS), Positive and Negative Affect (PANAS), Rumination-Reflection Questionnaire (RRQ) a measure of Self-Efficacy using the State Hope Scale (SHS), and a questionnaire to assess general health (SF-36). In order to subjectively assess the degree to which respondents have appraised situations in their life to be stressful on a given day, we will administer the 14 item PSS. VI patients have reported transient episodes of vision loss or reductions in visual fields that are attributed to stress; therefore, the PSS will be measured on a weekly basis. The PANAS questionnaire consists of 10 positive affect items, which reflect one’s level of pleasurable engagement with the environment, and 10 negative affect items, which are a general factor of subjective distress. The RRQ is a 12-item scale to measure aspects of self-focus, rumination and reflection. The SHS is a brief, 6-item scale to assess goal directed thinking at a given moment in time. The SF-36 is comprised of 36 questions and yields an 8-scale profile of functional health and well-being scores. The POMS-SF is a 30-item scale used to measure affective mood states rated on a 5-point Likert scale (0=Not at all, 4 = Extremely) during the past week including today [^15^](#_ENREF_15). The self-report questionnaire provides a global Total Mood Disturbance score as well as six affective dimensions subscales: *Tension/Anxiety, Depression/Dejection, Anger/Hostility, Vigor/Activity, Fatigue/Inertia,* and *Confusion/Bewilderment*. The questionnaires have been previously validated in other populations, are reliable and routinely used for clinical and research purposes. The questionnaires will be available on line and should take about 45 minutes for the subjects to answer all questionnaires.

**Balance and FOF**

***Fear of Falling Questionnaire****:*We will assess FoF 2 ways[^14^](#_ENREF_14). First, we will ask a single yes/no question, “In general, are you worried or afraid you might fall?” (The question is asked 3 times, specified as “at home,” “out of the home,” or “in the community.”). A single question regarding FoF has been found to have high test-retest reliability and high concurrent validity with continuous measures of FoF[^16^](#_ENREF_16). Second, we will administer a brief questionnaire to determine the prevalence of FoF in our population. The *Illinois Fear of Falling Scale (IFFS)* consists of 16 activities that might be avoided due to Fear of Falling. Subjects are asked to rate each item on a 4-point scale, indicating how much worry accompanies the performance of these activities. The IFFS was developed using a Rasch analytic model, which allows for objective scaling of questionnaire responses, and has been used with glaucoma patients at the Wilmer Eye Institute. It was found to substantiate the association between fear of falling and visual field loss.

**Balance Tests**

***Static Balance WBB tests****:* The next sequence of tests will be performed on a WBB. The WBB will be used to measure Center of Pressure (COP), derived from data sent by the 4 force plate sensors in the WBB. Changes of the COP in the anterior-posterior (A-P) and the medial-lateral (M-L) directions will be derived from the stream of sensor data sent to a laptop via Bluetooth. The root mean square (RMS) of the CoP amplitude, sampled over a 60 (two-leg stance) or 30 (one-leg stance) period is used as a measure of postural instability allowing us to determine postural deficits experienced by the subjects.

The *Timed One-Leg Stance* protocol requires the subject to raise one foot off the ground. Time up to 30 seconds is recorded. Arms stay down at sides. Any touch of foot or hand to a surface stops the timer. The task is performed 3 times and the best of 3 is chosen. The ability to stand on a single-leg is an important predictor of falls in the elderly. It has been reported that individuals increase their chances of sustaining an injury due to a fall by two times if they are unable to perform a One-Legged balance Test for five seconds[^17^](#_ENREF_17). The performance of this test should take a maximum of 5 minutes. The COP RMS will be computed as it may provide clinically meaningful information.

The *modified Clinical Test of Sensory Interaction on Balance (mCTSIB)* evaluates how well the participant is using sensory – kinesthetic (firm or unstable surface) and visual (eyes open or closed) – input when one or both systems are compromised. Subject’s feet are placed so that the inner edges of both feet are one foot length (their own) apart, in four sensory conditions: standing on a firm (WBB) surface with eyes open (EO); standing on a firm surface with eyes closed (EC); standing on an unstable (foam + WBB) surface with EO; and standing on an unstable surface with EC. Subjects will fixate binocularly at a fixation point on a white wall in front (or in that general direction with head neutral if severely impaired); arms are crossed over the chest. In addition, the subjects will be asked to voluntarily sway as far as possible in the A-P and M-L directions and hold the posture at the end position without having to take a step. This gives an indication of the stability margins of the subjects. Time up to 1 minute is recorded. The task is performed three times and the best of three is chosen. Instability in any condition suggests a sensory problem. For this test, test-retest reliability was found to be 0.99, and inter-rater reliability was found to be 0.68-1.0. This task should take no longer than 20 minutes to complete.

**Physical Function**

Body Mass Index (BMI) (i.e. weight and height) will be determined at baseline and post-intervention.

Systemic blood pressure (using a digital monitor with an automated cuff) and pulse rate will be measured at baseline.

Subjects will then complete the *International Physical Activity Questionnaire (IPAQ),* a verbally administered, 7-item questionnaire pertaining to physical activity level. This questionnaire is short and should take less than 15 minutes to administer. It has been found to have a test-retest reliability of .79 in a normal adult population. This questionnaire classifies subjects on a 3-level scale: Low, Moderate, and High Activity. These scores are assigned based on detailed instructions provided by the IPAQ. Two assessors, who are masked to all other outcomes, will independently categorize each subject as having 'low', 'moderate' or 'high' activity levels.

The “Chair sit and reach test” is used to assess lower body flexibility, which is important for good posture, for normal gait patterns and for various mobility tasks, such as getting in and out of a bathtub or car. From a sitting position at front of chair, with leg extended and hands reaching toward toes, the number of inches (cm) (+ or -) between extended fingers and tip of toe will be measured[^18^](#_ENREF_18).

The “30-Second Chair Stand” is used to assess lower body strength, needed for numerous tasks such as climbing stairs, walking and getting out of a chair, tub or car. Also reduces the chance of falling. The number of full stands that can be completed in 30 seconds with arms folded across chest will be measured[^18^](#_ENREF_18).

**Respiratory Measures**

Respiratory rate will be measured twice for each participant to reduce within-session variability. It will be determined by counting the number of inhalations with a stethoscope for 30 seconds at rest and multiplying by two at baseline and post-intervention (Lim et al., 2002). (Normal adult rate is 12 to 18 breaths/minute).

**AYT *Treatment Validation***

Four measures will be collected to assess the benefits of the AYT, compliance, fidelity of treatment and evaluate the participant’s experience.

1. The *Philadelphia Mindfulness Scale* (PHLMS). This 20-item scale assesses two components of mindfulness, *acceptance* and *awareness*. Total scores on both subscales range from 20 to 100, higher scores reflect greater mindfulness. The PHLMS will be collected at baseline, during week 4 and post- intervention. The PHLMS shows good internal reliability as reported in clinical and non-clinical samples. Total scores on both subscales range from 20 to 100, higher scores reflect greater mindfulness. This scale was chosen because the two components are orthogonal, or independent. Interestingly, *acceptance* is associated with reduced depression, anxiety and rumination but is unrelated to *awareness.* Thus, the PLHMS will allow us to determine distinct benefits associated with *mindfulness* induced by the yoga intervention.

2. *Practice/Homework Logs.* Participants will be asked to complete a weekly log online to report the activity performed, time allotted to the practice and will be used to evaluate compliance.

3. *Treatment Fidelity.* The yoga sessions will be videotaped and a treatment observation log by an independent observer (e.g. research assistant) will be completed during 4-6 yoga sessions chosen randomly to confirm adherence to the yoga treatment.

4. *Exit survey.* Participants will complete a brief, anonymous survey regarding their experience with the AYT. The survey is comprised of open-ended and multiple-choice response items (e.g. treatment expectations).

**Intervention Phase**

*Ashtanga-based Yoga Therapy*. The AYT includes one session a week with the instructor (i.e. Dr. Jeter) and participants will additionally be asked to perform at least two home-based practice sessions per week for a total of 8 weeks. An orientation session will be conducted during the baseline period prior to the intervention to familiarize subjects with yoga basics that include: Alignment, Breathing, Set up, Supplies, Journaling, and Room Temperature. Several studies have successfully used an 8-week period that produced positive outcomes. Participants will be led through the sequence of postures and the instructor will make adjustments and answer questions as needed. Participants will be asked to continue a home-practice twice a week. An audio CD will be provided to facilitate the home practice and ensure duration and compliance to the sequence. All participants will be encouraged to keep a journal either on the computer or with the help of a sighted companion. Interactions with the instructors and keeping a journal are meant to encourage compliance. Intervention sessions will be held in small groups if possible, or individually if necessary due to scheduling issues.

*Study Outcome Measures.* All outcome measures will be administered at baseline and post-intervention. Mood questionnaires will additionally be measured during week 4. The PHLMS will be collected at 3 time points, baseline, week 4 and post-intervention. The online questionnaires administered weekly during the intervention will include the Perceived Stress Scale (PSS) and the Positive and Negative Affect Schedule (PANAS). Participants will be asked to complete a weekly practice logs online to report the activity performed, time allotted to the practice and will be used to evaluate compliance. Participants will complete a brief exit survey online regarding their experience with the AYT. The survey is comprised of open-ended and multiple-choice response items (e.g. treatment expectations).

1. ***Study duration and number of study visits required of research participants.***

The pilot study will be completed over the course of 4-6 months. The intervention phase will be conducted over the course of 12 weeks and will require 12 study visits. The baseline period will be used to collect the full Baseline Assessment Battery and assign the Sleep Diaries. The orientation session will be conducted on the same day as the first class. Post-intervention (i.e. after 9-weeks) will consist of a two-week period to collect the Post-Assessment Battery (all measures from baseline) and assign the Sleep diaries. The waitlist control will begin the yoga treatment after pre- and post- measures have been obtained during the first round of testing. The final set of post-tests after the waitlist group has completed the 8-week yoga class, will allow us to compare effect of AYT on the two groups, and determine retention of any gains from the first AYT block.

1. Blinding, including justification for blinding or not blinding the trial, if applicable.

N/A

1. Justification of why participants will not receive routine care or will have current therapy stopped.

N/A

1. Justification for inclusion of a placebo or non-treatment group.

All enrolled participants will eventually receive the AYT intervention.

1. Definition of treatment failure or participant removal criteria.

Subjects may be removed from the study if they demonstrate lack of cooperation by repeatedly missing appointments, or by providing highly inconsistent responses that suggest lack of concentration.

1. Description of what happens to participants receiving therapy when study ends or if a participant’s participation in the study ends prematurely.

There are no consequences to participants if they leave the study early or are removed from the study for non-compliance.

1. **Inclusion/Exclusion Criteria**

Inclusion criteria:
- Age 18+

- Legal blindness (visual acuity worse than 20/200 but better than no/bare light perception, and/or visual field less than 20º, in the better eye)

- Any ocular disease diagnosis that is expected to remain relatively stable throughout a 3-6 month period

- Being healthy to the extent that participation yoga therapy would not exacerbate any existing disease conditions;

- Willingness to participate on a weekly basis for the 12-week intervention;

Exclusion criteria:

- Significant changes to vision within the most recent 3-month period

- Inability to understand study procedures or communicate responses to visual stimuli in a consistent manner

-Vestibular problems

-Symptomatic cardiovascular disease

-poorly controlled blood pressure

-history of neurologic disease

-acute orthopaedic problems that affect ambulation

-metastatic cancer

-pregnant women

1. **Drugs/ Substances/ Devices**
2. The rationale for choosing the drug and dose or for choosing the device to be used.

N/A

1. Justification and safety information if FDA approved drugs will be administered for non-FDA approved indications or if doses or routes of administration or participant populations are changed.

N/A

1. Justification and safety information if non-FDA approved drugs without an IND will be administered.

N/A

1. **Study Statistics**
2. Primary outcome variable.

The primary outcome variables are objective and subjective measures of sleep and balance for participants with low vision.

1. Secondary outcome variables.

Secondary measures include FOF, psychosocial mood, AYT validation, physical function and respiratory measures across all subjects.

1. Statistical plan including sample size justification and interim data analysis.

Baseline demographics and clinical variables will be summarized and compared for the 2 groups using descriptive statistics and parametric or non-parametric tests as appropriate. *Primary analysis:* Multiple regression analyses will determine the relationship between vision loss and sleep disturbances. We hypothesize that measures of vision function will be the best predictors of sleep disturbance after accounting for age, general health and psychosocial factors.

Group differences (e.g. age, level of fitness) will be used as covariates in the main intervention analysis. Group differences during post-intervention assessment will be estimated using a multivariate analysis of covariance (ANCOVA), with the baseline measures as covariates. Groups will be compared for the primary, as well as secondary measures. *Secondary Analyses:* A multiple regression analysis will be conducted to determine whether impaired visual function (VA, CS, VF) can predict the outcomes for static and FoF while controlling for age and fitness. A post-hoc analysis will determine whether subgroups (e.g. congenital blindness vs. acquired blindness) or other prognostic factors can reliably predict outcome measures. To assess the AYT, items from the exit survey will be evaluated and summarized to reveal patterns in the subjects’ overall experience. Practice logs will give us information related to compliance, total practice time during the week, and progress in terms of difficulty over the course of the 8 weeks. Treatment fidelity is determined by AYT adherence as measured by the observation log.

A sample size calculation is not applicable to this type of exploratory, pilot study, however, a minimum of 10 subjects is feasible to provide an indication of the acceptability of the AYT intervention and to help suggest possible benefits across a moderate-sized group of visually-impaired subjects. It will also allow us to further develop the intervention protocol and plan for larger scale RCTs as indicated.

1. Early stopping rules.

N/A

1. **Risks**
2. Medical risks, listing all procedures, their major and minor risks and expected frequency.

The vision tests we plan to evaluate do not entail any risks and we do not anticipate any study related adverse events or injuries related to them. In the case that a subject experiences a significant change in vision between visits, a dilated eye examination will be performed in order to determine the cause of the change, and/or appropriate referrals will be made as needed.

To minimize the discomfort associated with completing study questionnaires and diaries, subjects will be informed that they are free to refrain from answering any questions that make them uncomfortable of that they perceive as being personal or sensitive and do not wish to share as part of the study. If information gathered through the research indicates that a participant needs urgent psychiatric care (e.g. expressed suicidality), Drs. Dagnelie and Bittner will be immediately notified and the subject will be referred for immediate care.

Prior to the intervention, participants will notify the instructor of all pre-existing physical injuries.

The instructor will note each condition for each subject and suggest modifications for each pose based on subjects’ responses. To reduce the risk of injury, subjects will be advised to never force or strain themselves during exercise. If subjects feel pain, they will be advised to stop immediately and seek medical attention if necessary.

Testing of physical abilities through Balance and Physical function tests pose a very small possibility of falls or injury. However, subjects who feel uncomfortable can opt out of the balance tests. Research coordinators administering the tests are trained to identify individuals who may be at risk of injury during testing and will not perform tests on subjects for whom they feel testing would be unsafe. Coordinators will be trained in methods to prevent injury in cases where a subject loses his/her balance or begins to fall. No injuries have occurred in over 200 individuals tested with this protocol in other studies at the Wilmer Eye Institute and in over 10,000 subjects studied in other protocols[^19^](#_ENREF_19).

Another risk that requires management is the potential breach of patient confidentiality. The study investigators have been trained in the methods necessary to protect subjects’ privacy and every attempt will be made to maintain records and data in secure databases and areas. This study poses minimal additional risk. The societal benefits outweigh these risks.

1. Steps taken to minimize the risks.

If dilating drops are used, study personnel are trained in detecting ophthalmic adverse effects, including acute glaucoma.

1. Plan for reporting unanticipated problems or study deviations.

In the very unlikely event that a serious adverse event occurs, the study co-investigator(s) will immediately notify the IRB within 24 hours of the occurrence, using the standard JHU Adverse Event report form.

1. Legal risks such as the risks that would be associated with breach of confidentiality.

N/A

1. Financial risks to the participants.

N/A

1. **Benefits**
2. Description of the probable benefits for the participant and for society.

There will be no direct benefit to the subjects as a result of performing the vision tests, questionnaires, or balance tests. We will share the results of the vision tests with the subjects. At the end of the study, subjects’ visual status will be monitored for any significant changes by a licensed clinician, which may be considered a benefit. Any new information regarding the subjects’ vision may be shared with their eye care provider, at the subjects’ request.

Potential benefits may occur during the yoga intervention; however, this has not been previously demonstrated with scientific study among this patient population. Other types of patient populations have experienced decreased stress, anxiety, fatigue, depression, improved balance, and sleep as a result of yoga interventions. The anonymous exit surveys may allow the subjects to learn more about the experiences of other visually impaired subjects as a result of their AYT practice.

1. **Payment and Remuneration**
2. Detail compensation for participants including possible total compensation, proposed bonus, and any proposed reductions or penalties for not completing the protocol.

Participants will receive a free yoga mat and AYT instruction, as well as parking validation if they travel to our center with a sighted companion.

1. **Costs**
2. Detail costs of study procedure(s) or drug (s) or substance(s) to participants and identify who will pay for them.

The subjects will not be responsible for any costs directly related to the project, with the exception of transportation-related costs to our center.

Funding is provided by a scholarship from the Delta Omega public health honor society and by the Mona Wong Chou Wilmer Research Grant.

**REFERENCES**
