# Supplementary material for: Ashtanga-Based Yoga Therapy Increases the Sensory Contribution to Postural Stability in Visually-Impaired Persons at Risk for Falls as Measured by the Wii Balance Board: A Pilot Randomized Controlled Trial
Source: PLoS One. 2015 Jun 24;10(6):e0129646. doi: 10.1371/journal.pone.0129646 (PMC4479589; doi:10.1371/journal.pone.0129646)

**S1 Appendix. Normally-sighted versus visually impaired stability indices**

The data set for the normally-sighted individuals has been published elsewhere as part of a separate study to establish reliability of the WBB and therefore, we calculated the SI from the normal data for the purpose of comparison with the SI for VI in the current paper (Figure A). Only baseline data is available for the normally-sighted. Comparing baseline SI from normally sighted individuals to post-AYT VI using independent samples t-tests indicate no difference between groups (SI_EC_: t(28) = 0.51, p = 0.62, SI_VE_: t(28) = -1.37, p = 0.18), suggesting that after AYT training the VI group approached the normally-sighted baseline. We did find a significant difference between normal and VI for SI_EO_; however, this is to be expected as the normally-sighted group had full use of visual sensory information in EO conditions. The somatosensory contribution in the VI group is significantly greater than in the normal group, suggesting a greater use of somatosensory information.

**Figure A. Sensory contribution of normally-sighted versus visually impaired stability indices**


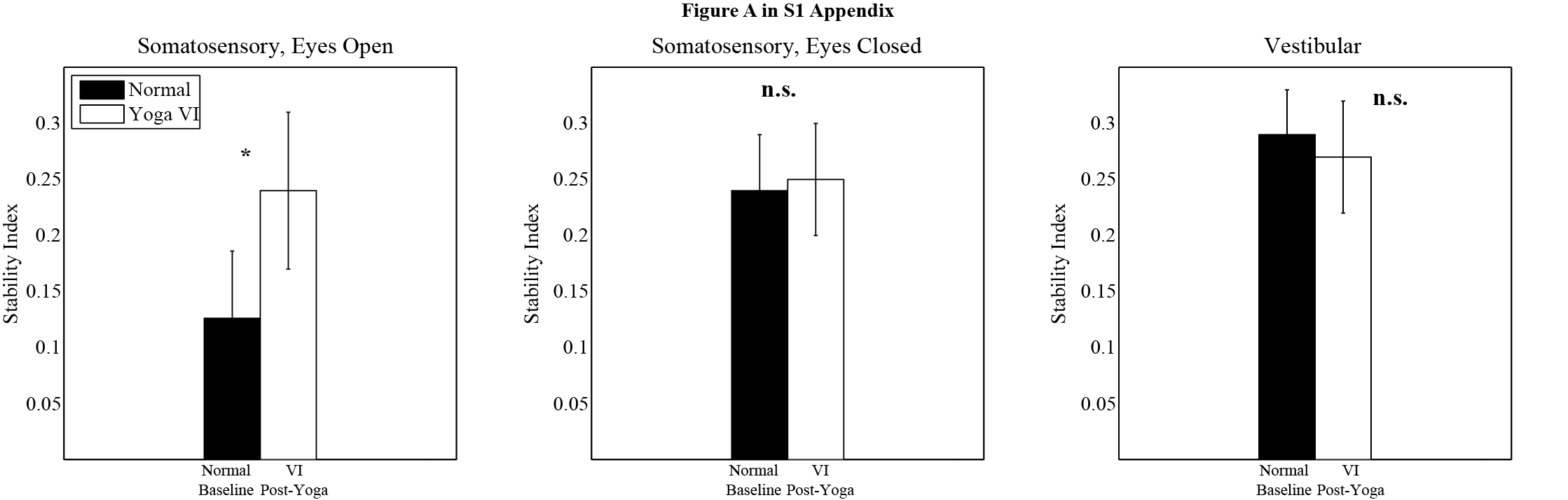

Supplement: S1 Appendix — (DOCX) [file pone.0129646.s003.docx]
